# 

**Published:** 1847-01

**Authors:** 


### BRITISH AND FOREIGN MEDICAL REVIEW,

OR

### Quarterly Journal

OF

### PRACTICAL MEDICINE AND SURGERY.

EDITED BY

JOHN FORBES, M.D. F.R.S. F.G.S.

FELLOW OF THE ROYAL COLLEGE OF PHYSICIANS IN LONDON;

Honorary Member of the Cambridge Philosophical Society, of the Academy of Sciences at Madrid, of the American Philosophical Society; of the Imperial and Royal Society of Physicians of Vienna, of the Royal Society of Göttingen, of the Royal Medical Society of Copenhagen, of the Medico-Chirurgical Society of Amsterdam, of the Medico-Chirurgical Society of Turin, of the Royal Swedish Medical Society, &c. &c.; Physician in Ordinary to Her Majesty's Household; Physician Extraordinary to His Royal Highness Prince Albert; Physician in Ordinary to His Royal Highness the Duke of Cambridge; and Consulting Physician to the Hospital for Consumption and Diseases of the Chest.

No. XLV.

JANUARY, 1847.

LONDON:

JOHN CHURCHILL, PRINCES STREET, SOHO;

SMITH, ELDER, & CO. CORNHILL.

[illegible] AND J. ADLARD, BARTHOLOMEW CLOSE.

